# International Congress

**Published:** 1886-06

**Authors:** 


### EDITORIAL.

### International Congress.

The following from the proceedings of the American Medical Association, recently held in St. Louis, will be of interest to the members of the dental profession, as indicating that progress is being made, preparatory to the meeting of the International Medical Congress.

Entire harmony prevailed in the Association upon this subject. The disaffected were either not present, or have ceased opposition, or may we not hope have come to see things differently than heretofore, and will give aid and co-operation to make the Congress a success.

### PRELIMINARY ORGANIZATION OF THE INTERNATIONAL MEDICAL CONGRESS OF 1887.

Your committee on the preliminary organization of the Ninth International Medical Congress, to be held in Washington, D. C., in 1887, have the honor to respectfully report:

That your committee, in accordance with your instructions, after mature deliberation, adopted the necessary rules for the organization; nominated general officers for the Congress and its Sections, and a local committee of arrangements at Washington, with power to increase its membership, and in accordance with Rule 10, as heretofore published in THE JOURNAL of the Association, they have constituted the proposed officers of the Congress, and the Presidents of its Sections, as an Executive Committee, for the further prosecution of the work of organization, and your committee herewith submit a list of the members of the organization.

OFFICERS OF THE CONGRESS.

*President*—Nathan S. Davis, of Chicago.

*Vice-Presideuts*—Wm. O. Baldwin, of Alabama; Wm. Brodie, of Michigan, ; W. W. Dawson, of Ohio; J. A. Grant, of Ottawa, Canada; E. M. Moore, of New York; Tobias G. Richardson, of Louisiana; Lewis A. Sayre, of New York; J. M. Toner, of Washington, D. C.; The President of the American Medical Association; The Surgeon-General U. S. Army; The Surgeon General U. S. Navy; The Supervising Surgeon General U. S. Marine Hospital Service.

*Secretary-General*—John B. Hamilton, of Washington, D. C.

*Treasurer*—E. S. F. Arnold, of New York.

*Chairman of the Finance Committee*—Richard J. Dunglison, of Philadelphia, Pa.

PRESIDENTS OF SECTIONS.

*General Medicine*—A. B. Arnold, of Baltimore, Md.

*General Surgery*—Wm. T. Briggs, of Nashville, Tenn.

*Military and Naval Surgery*—Henry F. Smith, of Philadelphia, Pennsylvania.

*Obstetrics*—De Laskie Miller, of Chicago, Ill.

*Gynæcology*—James F. Harrison, of the University of Virginia.

*Therapeutics and Materia Medica*—F. H. Terrill, of San Francisco, Cal.

*Anatomy*—Wm. H. Pancoast, of Philadelphia, Pa.

*Physiology*—J. H. Callender, of Nashville, Tenn.

*Pathology*—A. B. Palmer, of the University of Michigan.

*Diseases of Children*—J. Lewis Smith, of New York, N. Y.

*Ophthalmology*—E. Williams, of Cincinnati, Ohio.

*Otology*—S. J. Jones, of Chicago, Ill.

*Laryngology*—W. H. Daly, of Pittsburgh, Pa.

*Dermatology and Syphilis*—A. R. Robinson, of New York City.

*Public and International Hygiene*--Joseph Jones, of New Orleans, La.

*Collective Investigation, Vital Statistics, and Climatology*--Albert L. Gihon, U. S. Navy, Washington, D. C.

*Psychological Medicine and Nervous Diseases*--John P. Gray, of Utica, N. Y.

*Dental and Oral Surgery*--Jonathan Taft, of Cincinnati, Ohio.

On motion of Dr. A. L. Gihon, U. S. N., it was *unanimously* accepted and adopted.

Dr. Henry H. Smith, Pa., offered a motion to reconsider this vote.

On motion of A. L. Gihon, this motion was laid on the table.

ENTERTAINMENT OF THE INTERNATIONAL CONGRESS IN 1887.

On motion of A. Y. P. Garnett, seconded by Dr. D. W. Yandell, it was

*Resolved*, That the delegates to this Association be requested upon their return to their homes to adopt such means as may to them seem best to call the attention of their respective delegates to the Congress of the United States to the desirability of making an appropriation to assist the medical profession of this country in properly receiving and entertaining the International Medical Congress in Washington in 1887.

### The International Medical Congress.

The following, from an editorial in the *British Journal of Dental Science*, of May 1st, shows quite conclusively, that the dentists of England have a good degree of interest in the success of the International Medical Congress, to be held next year. A warm interest is expressed in the Congress, even if there were no dental section, but this being established, affords additional interest, and we are assured that there will be a large attendance from abroad:

"Whatever may be the fate of the Dental and Oral Section of the Congress, it seems more than probable that a most influential meeting will take place in 1887. We heard of names as likely to hold office which must insure the respect and wide support of English surgeons and physicians. It will be a sad miscarriage if American dentists allow internecine conflicts to mar the chance of promoting a brilliant and effective dental section. Whatever may be said, there is no doubt that in Britain the medical and dental professions are so allied in interests and pursuits that the English dentists will be much more disposed to go over to America to attend a Dental Section of an International Medical Congress than they would be to put themselves out of the way to appear at an International Dental Congress. The Medical Congresses are *faits accomplis*, not so the Dental, at least in the eyes of the dental profession in England, so if we are to take part in the meetings of American dentists it will have to be at the Medical Congress. Many dentists will go across whether there is a section or not and will attend the Medical Congress. We will, therefore, trust that Dr. Taft's circular will carry out its assurances, and we may all meet and compare notes before a brilliant Dental and Oral Section of the Ninth International Medical Congress. Such there certainly could be."

